# NICE: A Two‐Step Non‐Invasive Framework for Embryo cfDNA Read Enrichment and Quality Assessment

**DOI:** 10.1002/advs.77327

**Published:** 2026-08-24

**Authors:** Xueya Zhou, Shu Ding, Zhenyi Zhang, Qiaoling Shangguan, Jie Qiao, Peijie Zhou, Yidong Chen

**Affiliations:** ^1^ Center for Machine Learning Research Center for Quantitative Biology State Key Laboratory of Female Fertility Promotion Center for Reproductive Medicine Department of Obstetrics and Gynecology Third Hospital Peking University Beijing China; ^2^ AI for Science Institute Beijing China; ^3^ National Clinical Research Center for Obstetrics and Gynecology Beijing China; ^4^ School of Mathematical Sciences Peking University Beijing China; ^5^ Peking‐Tsinghua Center for Life Sciences, Academy for Advanced Interdisciplinary Studies Peking University Beijing China; ^6^ National Engineering Laboratory for Big Data Analysis and Applications Beijing China

**Keywords:** assisted reproductive technology, computational biology, genetic testing, machine learning, preimplantation genetic diagnosis

## Abstract

Non‐invasive preimplantation genetic testing (niPGT) using cell‐free DNA (cfDNA) extracted from spent embryo culture medium (SECM) has shown great potential, providing economic and practical advantages for embryo ploidy testing and quality assessment, while minimizing the risk of embryo damage. However, maternal DNA contamination, which can result in sex discordance and false‐negative findings, remains a critical barrier to its clinical application in embryo prioritization. In this study, we present the NICE (Non‐Invasive CfDNA‐based Embryo assessment) framework, designed to enable contamination‐resistant evaluation and support accurate embryo selection. The workflow integrates a two‐step strategy: first, embryonic cfDNA is effectively purified using DECENT‐plus—an enhanced version of our previously established deep CNV reconstruction algorithm (DECENT)—that minimizes interference from polar body‐derived maternal DNA and improves signal resolution between maternal and embryonic origins. Subsequently, machine learning models based on biometric features extracted from the purified cfDNA are constructed to classify embryo quality, providing intelligent decision support for clinical embryo prioritization. By addressing the challenge of maternal contamination, NICE establishes a more automated, standardized and non‐invasive paradigm for embryo quality assessment and underscores the critical role of cfDNA‐based analysis in facilitating non‐invasive selection of high‐quality embryos to improve outcomes in assisted reproductive technology.

## Graphical abstract

The non‐invasive NICE framework, built on an ensemble stacking machine learning model, prioritizes embryos by analyzing cell‐free DNA from spent culture medium. By integrating multimodal signals, including genomic and epigenetic profiles, this automated approach standardizes morphological assessment without human bias, paving the way for more precise and objective embryo selection.

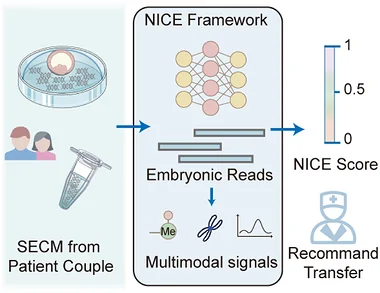

## 1 Introduction

In assisted reproductive technology (ART), accurate assessment of embryo quality is fundamental to improving in vitro fertilization success rates. Current clinical embryo selection strategies primarily rely on morphological evaluation, which classifies embryos based on parameters such as cell number, cleavage rate, and degree of fragmentation [1, 2]. As the labeling basis for this study, morphological grading provides reliable phenotypic labels for subsequent feature analysis. However, morphological assessment primarily reflects the external characteristics of embryos and fails to provide biological information regarding their genetic status, while also being subject to inter‐operator variability. To overcome the limitations of traditional approaches, researchers have begun integrating multiple techniques for embryo evaluation, including metabolic data analysis, novel morphological grading systems, digital imaging, and preimplantation genetic testing (PGT), etc [3, 4, 5, 6]. PGT optimizes embryo selection through comprehensive screening of chromosomal abnormalities, single‐gene disorders, and structural rearrangements, minimizing genetic risks in assisted reproductive technology [6]. Nevertheless, PGT is constrained by high costs, procedural complexity, invasive sampling, and potential misdiagnosis due to mosaicism [7].

Currently, there is a critical demand for a clinically practical, non‐invasive, yet highly accurate and objective method for embryo assessment. Recent progress in reproductive medicine has introduced non‐invasive preimplantation genetic testing (niPGT) based on cell‐free DNA (cfDNA) analysis from spent embryo culture medium (SECM) [8, 9]. DNA methylation, a key epigenetic regulator of embryonic development, not only provides insights into developmental competence but also allows precise tracing of cfDNA origins, making it invaluable for applications in disease diagnostics, prenatal screening, and transplantation monitoring [10, 11, 12]. Furthermore, the integration of DNA methylation markers into invasive PGT procedure enables discrimination between high‐ and low‐quality embryos, providing an additional parameter for embryo selection [13, 14]. Therefore, the integration of niPGT with DNA methylation profiling offers a promising non‐invasive approach for comprehensive embryo evaluation, combining ploidy detection with developmental potential assessment while maintaining superior embryo safety and cost‐effectivenesss, providing more accurate decision support for assisted reproductive technology.

However, a major challenge of this technology lies in the contamination of maternal DNA in SECM. The cfDNA in SECM mainly originates from the trophoblast ectoderm (TE), inner cell mass (ICM), cumulus cells and polar bodies (PBs) [15, 16]. Removing maternal contamination helps to reduce the false negative rate and the rate of gender inconsistency. The existing strategies for reducing pollution levels mainly focus on the improvement of the sample processing procedures [17, 18, 19]. However, these methods have limited effectiveness in eliminating parent contamination and have greatly increased the workload. Therefore, our previous work proposed deep copy number variations (CNV) reconstruction (DECENT), an innovative machine learning framework designed to mitigate maternal cumulus cell contamination in SECM while reconstructing embryonic CNV [20]. DECENT integrates both sequence and methylation characteristics from embryonic and maternal sources, leveraging convolutional neural networks (CNNs) combined with attention mechanisms to precisely determine the cellular origin of sequencing reads. However, DECENT was specifically designed to eliminate maternal cumulus cell contamination, and to date, no existing algorithm in the field has been capable of removing maternal PBs contamination from culture medium.

To address this critical gap, we developed Non‐Invasive CfDNA‐based Embryo assessment (NICE), a two‐step strategy that first purifies embryonic cfDNA and then extracts key features for accurate morphological quality classification. First, we further removed the maternal contamination from the cumulus cells and PBs and extended the input sequence length from 66 bp (for DECENT) to 132 bp by constructing DECENT‐plus. The ability of the model to distinguish maternal (cumulus cells/PBs) and embryonic (TE/ICM) origin cfDNA has been further improved. Next, we extracted biological characteristics solely based on cfDNA from embryos and constructed a machine learning model for embryo quality classification, and demonstrated its potential application in embryo selection. This strategy aims to remove maternal contamination in SECM, which can be used to improve the diagnostic accuracy of niPGT and combined with morphology for screening high‐quality embryos.

## 2 Results

### 2.1 NICE Framework for Embryo Quality Assessment

**FIGURE 1:**
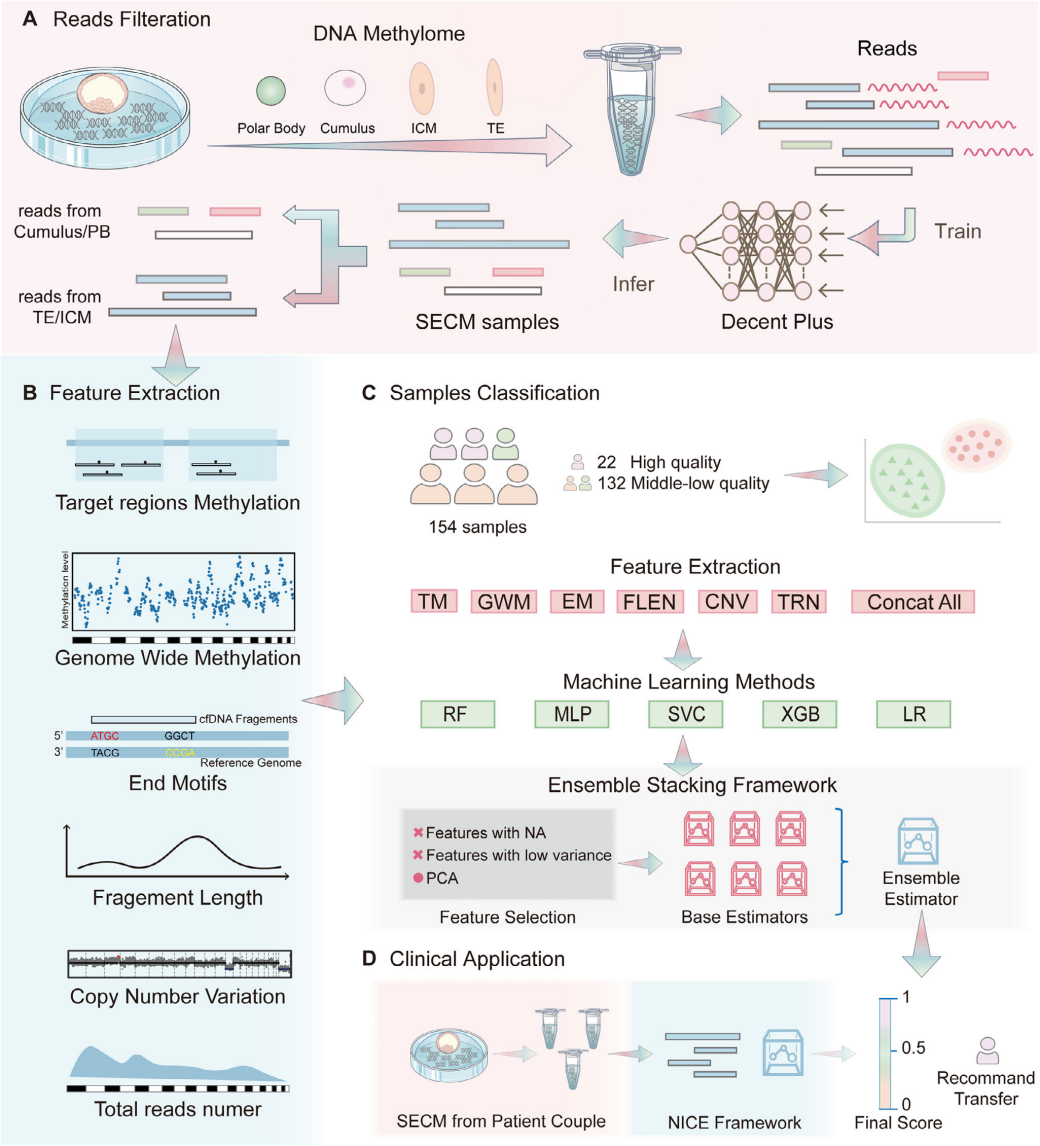
Workflow for embryo quality classification using cfDNA methylation sequencing data from spent embryo culture medium (SECM). (A) A deep‐learning based algorithm DECENT‐plus was trained on single‐cell methylation reads from four cell types and used to infer cell‐of‐origin in SECM samples, to filter out maternal reads. (B) Multiple features were extracted from the embryo‐derived reads. (C) Various machine learning models were developed and ensembled using these features to classify embryo quality, with model selection and evaluation performed via cross‐validation. (D) Clinical application of the NICE framework. In clinical settings, SECM samples from patient couples are analyzed using the trained NICE pipeline to produce a final embryo quality score, which can guide embryo selection and embryo transfer decisions.

To revolutionize non‐invasive embryo assessment, we developed NICE, an integrated machine learning framework that analyzes cfDNA from SECM. Our system simultaneously solves two fundamental challenges in niPGT: (i) effective elimination of confounding maternal DNA contamination (derived from both cumulus cells and PBs), and (ii) comprehensive decoding of embryonic cfDNA signatures, predominantly originating from TE and ICM lineages, to establish a multidimensional biomarker panel for precise embryo quality prediction (Figure 1).

First, we built upon our previous work to distinguish between maternal‐ and embryo‐derived reads by incorporating expanded sources of maternal contaminations (Figure 1A). We constructed a large‐scale training dataset comprising approximately 30 million sequencing reads derived from four representative cell types, including cumulus cells and PBs as maternal sources, and TE and ICM as embryonic sources (Table S1; Note S1 for detailed data descriptions). In this new version, DECENT‐plus, we also extended the input sequence length from 66 bp (used in DECENT) to 132 bp, which improved the model's ability to classify SECM cfDNA reads into maternal (cumulus cells /PBs) and embryonic (TE/ICM) origins. Using DECENT‐plus, we performed read‐level classification on cfDNA from SECM samples and enriched embryo‐derived reads for downstream analysis.

To comprehensively characterize the embryo‐derived cfDNA reads, we extracted multiple biologically informative features (Figure 1B). Specifically, Target regions Methylation (TM) was calculated as the average methylation level across predefined genomic regions associated with embryonic development (Table S2) [21, 22, 23, 24]. Genome Wide Methylation (GWM) profiled methylation levels across the entire genome using fixed‐size bins. End Motif (EM) features captured the frequency distribution of 4‐mer sequences at the 5' ends of cfDNA fragments [25]. Fragment Length (FLEN) distributions were quantified by profiling the frequency of different fragment lengths within each sample [26]. Copy Number Variation (CNV) profiles were derived using Ginkgo to detect large‐scale chromosomal aberrations (Methods) [27, 28]. Finally, Total Read Numbers (TRN) features were generated by binning the genome into 5 Mbp windows and normalizing the number of mapped reads within each bin using Z‐scores [29]. These complementary features were designed to capture both epigenetic and structural properties of embryo‐derived cfDNA, providing a rich foundation for downstream classification tasks.

As the final step, we performed embryo quality classification based on features extracted from embryo‐origin cfDNA reads (Figure 1C). Samples were categorized into high quality and middle‐low quality groups. For each individual feature type (TM, GWM, EM, FLEN, CNV, and TRN), as well as the concatenated feature set (Concat), we first removed missing values and low‐variance features, followed by principal component analysis (PCA) to reduce dimensionality. For each feature category, five machine learning algorithms, Random Forest (RF), Logistic Regression (LR), Multi‐Layer Perceptron (MLP), Support Vector Classifier (SVC), and XGBoost (XGB), were trained and evaluated via 5‐fold cross‐validation. The optimal algorithm for each specific feature type was then identified based on its predictive performance. Subsequently, these best‐performing models from different feature types were integrated into a stacking ensemble framework to further enhance the overall classification accuracy. This multi‐feature framework provides a robust strategy for non‐invasive classification of embryo quality using cfDNA methylation signals from SECM samples.

Overall, the NICE framework employs a two‐step approach for non‐invasive embryo quality assessment. It first performs enrichment of embryo‐derived cfDNA fragments from SECM samples to minimize maternal DNA contamination. Subsequently, it extracts biologically meaningful features from the purified cfDNA and applies machine learning models to classify embryo morphological quality (Figure 1D). By enabling accurate, damage‐free evaluation of embryo quality, NICE holds potential to support clinical decision‐making and enhance the selection of high‐quality embryos for transfer in ART.

### 2.2 Purification of Embryo‐Derived Reads

In our previous work, DECENT was trained using SECM samples with high and low levels of maternal contamination, treating the two groups respectively as maternal‐origin and embryo‐origin reads [20]. This model successfully identified clean embryo‐derived reads, which were then used for CNV reconstruction. However, DECENT only considered contamination from cumulus cells, while another potential source of maternal DNA, PBs, was not explicitly removed.

To address this limitation, we developed DECENT‐plus using similar model architecture as DECENT, but with an enhanced input length of 132 bp per read as well as the expansion of training datasets by incorporing various contamination sources (Methods). The model was trained on 30 million reads derived from four distinct cell types (cumulus cells, PBs, TE, and ICM). DECENT‐plus predicts the probability of each read originating from maternal cells, enabling estimation of the proportion of maternal contamination within each SECM sample.

**FIGURE 2:**
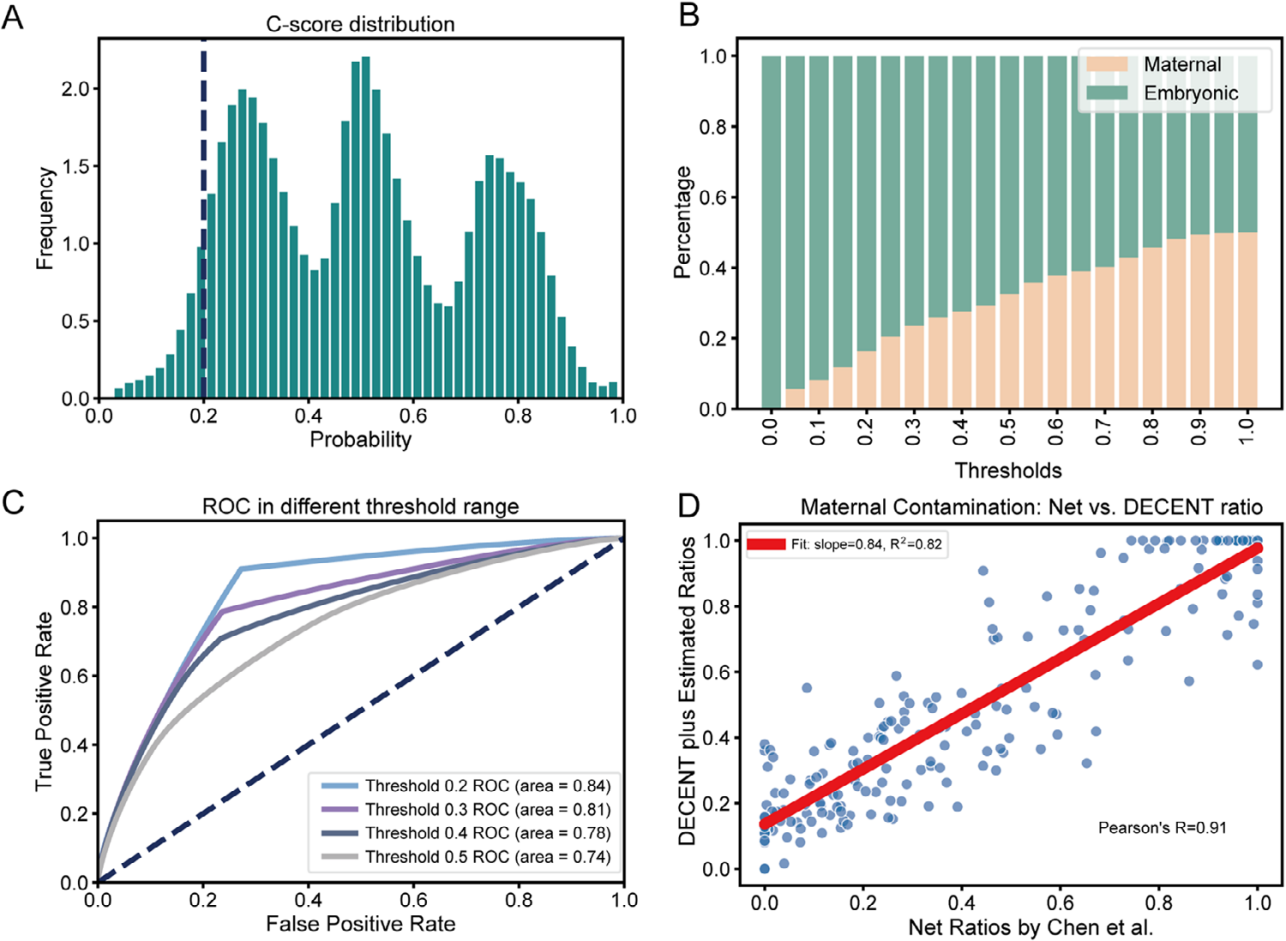
DECENT‐plus enables identification of embryo‐derived reads in SECM samples. (A) Distribution of DECENT‐plus predicted probabilities indicating maternal origin for each cfDNA read. (B) Stacked bar plots showing proportion of reads of the maternal and embryonic origin under different filtering thresholds. (C) ROC curves for embryo‐origin read classification under four different thresholds (0.2, 0.3, 0.4 and 0.5), demonstrating trade‐offs between precision and coverage. (D) Correlation of maternal contamination levels estimated by DECENT‐plus and the reported contamination levels from the original SECM study (Net ratio).

To evaluate the performance of DECENT‐plus, we split the dataset into training and testing sets with an 8:2 ratio. The trained DECENT‐plus achieved a classification accuracy of 67.54% on the test set, distinguishing between reads of cumulus cells/PBs and TE/ICM origin (Figure S1). We first examined the distribution of predicted maternal‐origin probabilities for reads in the test set (Figure 2A). The resulting histogram displayed a trimodal pattern, with peaks centered around 0.3, 0.5, and 0.7. Similar to observations from DECENT, a substantial number of reads exhibited intermediate probabilities near 0.5, indicating potential ambiguity in origin of cell due to shared genomic regions or overlapping methylation signatures between maternal and embryonic sources.

For downstream task, classifying embryo quality based on features from embryo‐derived reads, the model precision is prioritized over recall, so we aim to retain only highly confident embryo‐origin reads. Therefore, the selection threshold for read classification is critical: reads with predicted maternal‐origin probability below the threshold are classified as embryo‐derived, while those above are classified as maternal. A high threshold may result in retaining too many maternal reads, introducing noise into embryo quality classification. Conversely, a low threshold may retain too few reads for meaningful feature extraction. To determine an appropriate threshold, we assessed the distribution of maternal vs. embryo‐derived reads across a range of thresholds (Figure 2B). As the threshold increased, the proportion of reads classified as embryo‐derived significantly decreased. Notably, when the threshold was set to 0.2, approximately 80% of retained reads were predicted to originate from embryonic sources.

To refine threshold selection and further evaluate model performance, we excluded reads with intermediate probability values and focused on reads at the distribution extremes. We next benchmarked model performance at four probability thresholds (0.2, 0.3, 0.4 and 0.5) by computing the corresponding AUCs (Methods). Increasing the classification threshold led to a decrease in the model's accuracy, as evidenced by the continuous decline in the AUC value (Figure 2C). Peak performance (AUC = 0.84) was attained at the 0.2 threshold, underscoring DECENT‐plus's capacity to pinpoint embryo‐derived reads with high confidence.

Similar to DECENT, DECENT‐plus outputs a probability estimate for each read being derived from cumulus cells or PBs, enabling estimation of the overall maternal contribution in each SECM sample (Methods). In previous studies, maternal contamination levels were independently determined through methylation signature analysis, reported as net ratios representing the proportion of cumulus‐ and PB‐derived DNA [15, 16]. The DECENT‐plus estimates of maternal contamination were validated against the reference net ratios by correlation analysis (Figure 2D). Linear regression revealed a strong concordance between the two approaches (R^2^ = 0.62, slope = 0.84, Pearson correlation coefficient = 0.91), supporting the reliability of DECENT‐plus in deconvoluting cfDNA cellular origins in SECM samples. This consistency further supports the use of DECENT‐plus derived probabilities to substantially enrich embryo‐origin signals for downstream analysis, although it should be noted that this approach serves to minimize rather than completely eliminate maternal background contamination.

### 2.3 Filtered cfDNA Fragment Features Support Embryo Quality Classification

To evaluate whether embryo‐derived cfDNA reads from SECM samples filtered by DECENT‐plus contain biologically informative signals for embryo quality assessment, a set of fragmentomic and epigenomic features was extracted, including TM, GWM, EM, FLEN, CNV, and TRN. These features were computed per sample and used to train machine learning models for classifying embryo morphological quality. Samples with an AA morphological grade were labeled as high quality, whereas those with B^*^ or C^*^ grades were grouped as middle‐low quality (Methods).

**FIGURE 3:**
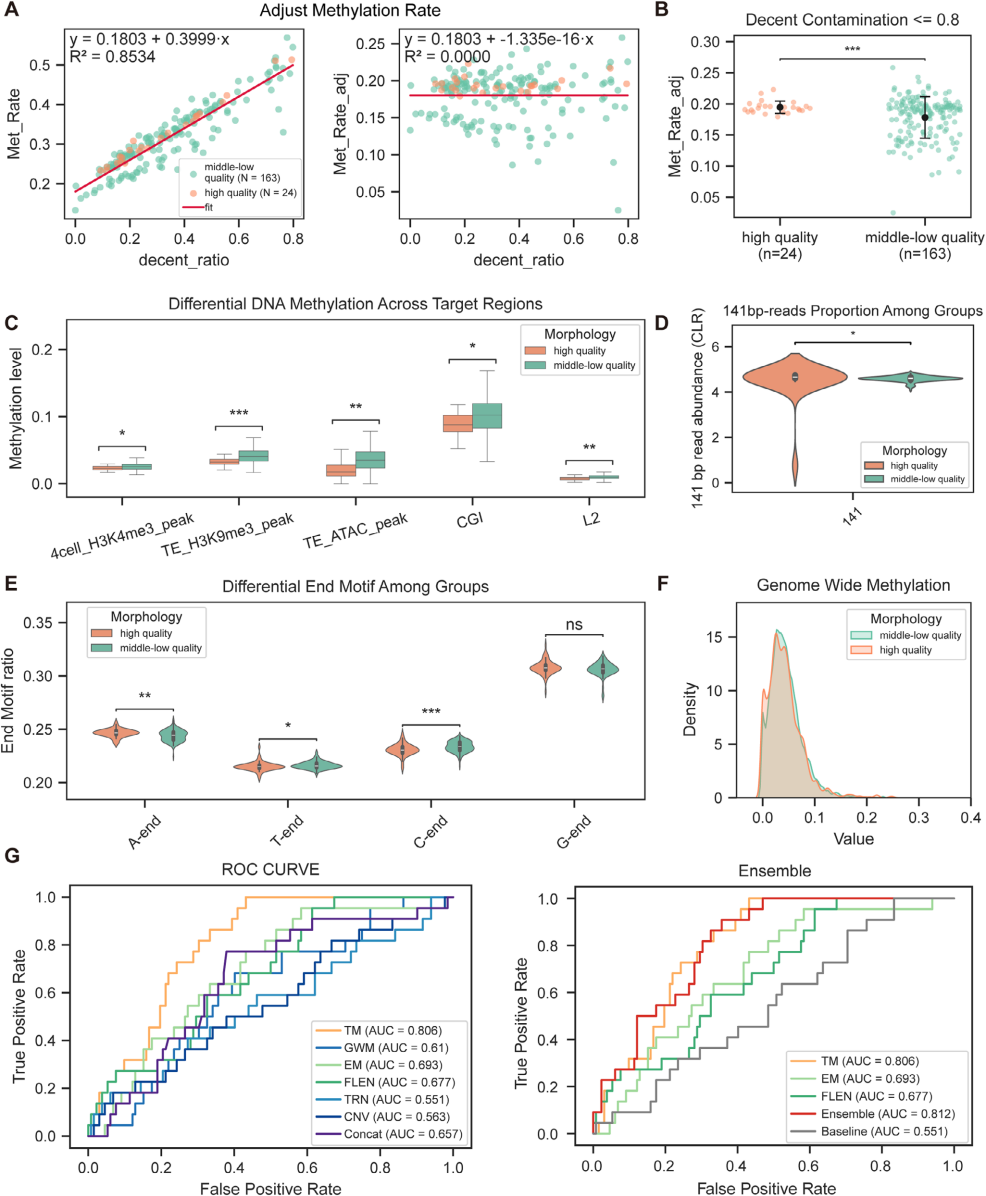
Embryo quality classification using embryonic‐derived cfDNA features and machine learning models. (A–F) Exploratory analysis of six categories of cfDNA features between middle‐low quality embryos (light green) and high quality embryos (orange). (A) Adjust methylation level based on sample marternal contamination level. (B) Difference of adjusted methylation level among different embyroic quality. (C) Boxplots of TM showing significant differences between groups. Statistical testing was performed using the Wilcoxon rank‐sum test. (D) Violin plot showing differences in the frequency of 141 bp FLEN reads between the two groups. Statistical testing was performed using the Wilcoxon rank‐sum test. (E) Violin plot showing differences in the frequency of EM between groups. (F) Density plot of GWM levels across groups. The Kolmogorov‐Smirnov test revealed a significant difference in distribution (*P* = 0.0365). (G) Cross‐validated performance evaluation of machine learning models. Models were trained on: (i) individual feature types, (ii) concatenated features (Concat), and (iii) a stacked ensemble (Ensemble) combining top‐performing models from each feature type. Performance was assessed through ten‐fold cross‐validation, with curves representing the combined predictions from all validation folds. Left: performance of models trained on each individual feature types. Right: performance of the stacked model (top 3 feature types) compared to the traditional SECM methylation rate‐based metric (Baseline). ^*^ *p* < 0.05; ^**^ *p* < 0.01; ^***^ *p* < 0.001.

Classification of embryo quality based on cfDNA methylation levels in SECM has been previously investigated [13]. However, high levels of maternal contamination were observed to artifactually increase apparent methylation levels. As shown in Figure 3A the maternal contamination level exhibited a significant positive correlation with the SECM methylation level. Accordingly, a simple linear regression was applied to adjust sample‐level methylation for contamination (Figure 3A). After adjustment, corrected methylation levels differed significantly across embryos of distinct morphological quality (Figure 3B), consistent with findings reported from direct embryo biopsy measurements (Figure S2) [14]. The corrected sample‐level methylation was subsequently included as one feature category within the TM feature set for classification.

In addition to sample‐level methylation, previous studies have shown that DNA methylation at specific genomic regions varies with developmental stages such as 2‐cell, 4‐cell, and morula, and that embryos of different morphological quality exhibit distinct methylation patterns in certain genomic region [14, 22]. Average methylation levels were therefore computed across 22 development‐associated regions and 24 genome‐based regions to define TM (Methods) [21, 22, 23, 24]. Several regions showed significant differences in methylation levels between high and middle‐low quality embryos (Figure 2C). For example, methylation in the 4‐cell associated region differed significantly between groups, as were regions such as CGI in hg19, in agreement with prior reports [14].

Fragmentomic features such as TRN and FLEN have demonstrated utility in cfDNA‐based cancer deconvolution and may also reflect embryo quality [29]. For TRN, the genome was divided into 5 Mb bins, read counts per bin were calculated and z‐score normalized was performed within each sample to account for sequencing depth. The bin‐wise mean TRN across embryo groups was further examined at a genome‐wide scale (Figure S3A). Overall, most genomic bins exhibited similar TRN distributions between groups, with only a limited number of regions showing modest differences. In terms of fragment size, a dominant peak at 141 bp was observed in SECM cfDNA. The frequency of 141 bp fragments differed significantly between the two embryo groups (Figure 3D).

The EM distribution, defined as the frequency of 4‐mer sequences at the 5’ terminal of cfDNA fragments, has been used to characterize nuclease cleavage patterns [25]. In maternal plasma, cfDNA end‐motif composition has been reported to associate with gestational diabetes [30]. Building on this rationale, we evaluated whether EM features can discriminate embryos of different morphological quality. The frequencies of all 256 possible 4‐mer motifs at the 5’ ends of reads were quantified, and several motifs exhibited significant between‐group differences (Figure 3E). In particular, the frequencies of fragments bearing A‐, T‐, or C‐ends, defined as fragments whose 5’ terminal nucleotide is A, T, or C, differed significantly between high quality and middle‐low quality embryos.

GWM was evaluated by dividing the genome into non‐overlapping 5 Mb bins and calculating average methylation levels per bin (Methods). Density plots showed that both groups contained a large number of bins with very low methylation levels (0–0.1) (Figure 3F). A Kolmogorov‐Smirnov test confirmed a significant difference in GWM distributions between the two groups. CNV has also been identified as a distinguishing feature of embryo quality [31]. PCA of CNV profiles revealed that high quality samples clustered more tightly, whereas middle‐low quality samples exhibited greater dispersion, indicating increased heterogeneity (Figure S3B).

The predictive power of these feature sets was next evaluated using machine learning. For each feature type, separate models were trained, as well as a model trained on the concatenated feature set. After removing missing values and low‐variance features, top features were selected by ANOVA F‐value for model training (Methods). Models were trained using five machine learning algorithms under five‐fold cross‐validation, with hyperparameters tuned via Optuna (Methods). For each feature type, the best‐performing model was selected as a base estimator, and prediction scores were integrated using an ensemble strategy. As shown in Figure 3G, although each of the six individual feature types demonstrated moderate classification performance, stacking the top three feature types (TM, EM and FLEN) using a voting ensemble enhanced predictive power. This ensemble achieved an AUC of 0.812, representing a substantial improvement relative to the traditional SECM‐derived methylation rate alone (AUROC = 0.551), which has been used as an empirical marker of embryo quality [13]. To further assess the necessity of the two‐stage NICE design, we performed a sensitivity analysis in which Stage one read‐level enrichment was omitted and all features were directly extracted from low‐contamination SECM samples. As summarized in Figure S4, models trained on raw cfDNA without DECENT‐plus enrichment consistently underperformed the full NICE pipeline at the overall level, highlighting the contribution of embryo‐derived read enrichment to robust embryo quality classification.

In summary, by extracting and modeling biologically meaningful fragmentomic and epigenomic features from embryo‐origin cfDNA, a robust non‐invasive approach for embryo quality evaluation based on SECM samples was demonstrated. The proposed pipeline may serve as a promising framework for embryo evaluation in ART in a non‐invasive manner.

### 2.4 Key Features Associated with Embryo Morphological Classification

**FIGURE 4:**
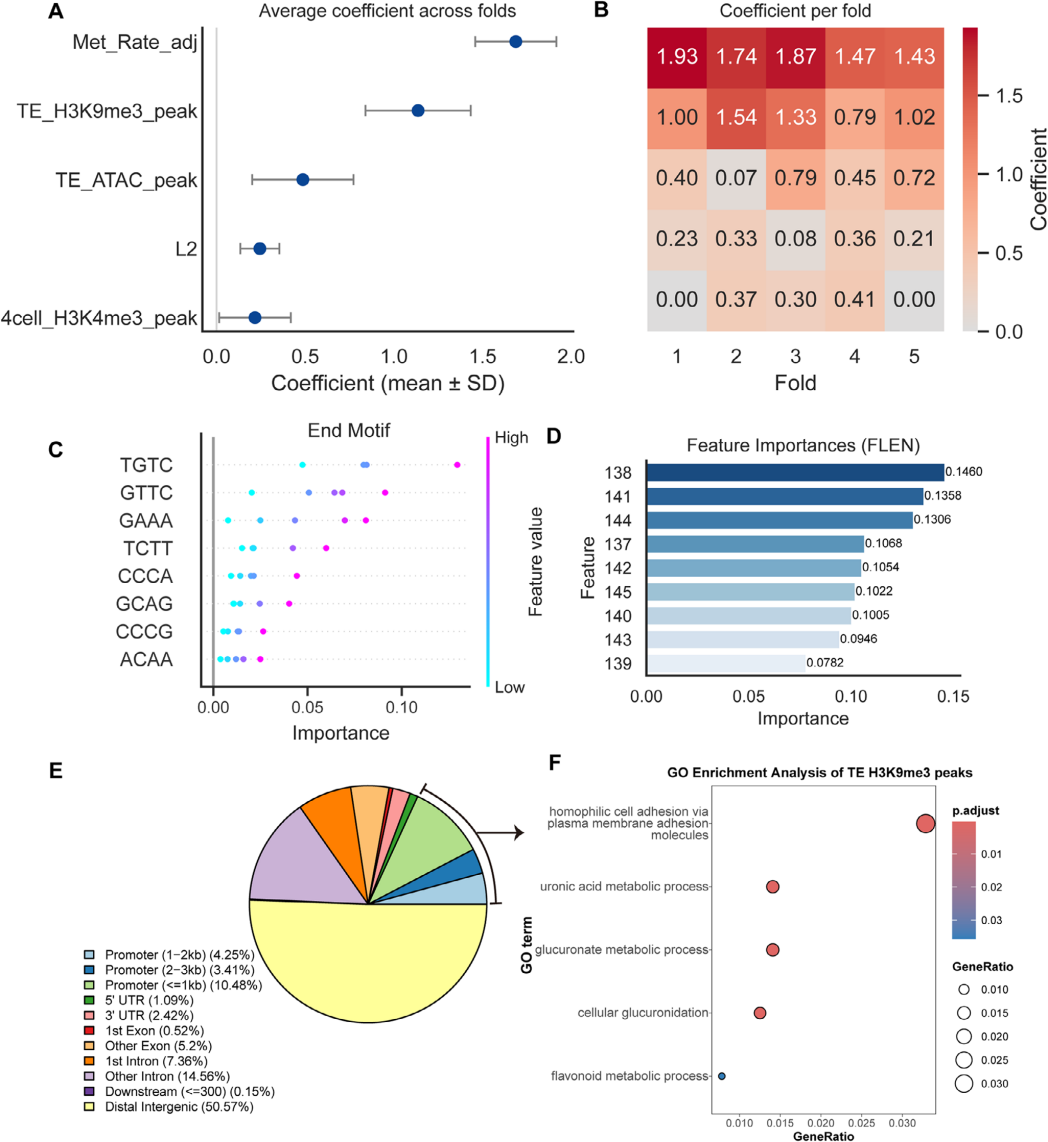
Interpretable analysis of machine learning models. (A) Top 5 most important features from the TM based model, averaged across five‐fold cross‐validation. (B) Heatmap of each feature in each fold. (C) SHapley Additive exPlanations beeswarm plot illustrating the contributions of the top EM features to the model predictions. (D) Barplot showing the most informative FLEN features ranked by model importance. (E) The distribution of annotated regions of TE H3K9me3 peaks (F) GO enrichment bubble plot for genes related promoter in E.

To identify the cfDNA‐derived features most effective for predicting embryo morphological quality and to enable biological interpretation of model outputs, interpretability analyses were undertaken to quantify feature contributions across models. Individual models based on TM, EM, and FLEN feature sets exhibited strong classification performance, with AUC values of 0.806, 0.693, and 0.677, respectively. Feature importance profiles were then examined to identify the most informative predictors. As shown in Figure 4A, B, the five most influential TM features, averaged across cross‐validation folds, consistently highlighted the adjusted sample‐level methylation as the dominant predictor of embryo quality. The average methylation level within TE H3K9me3 peak regions, where H3K9me3 denotes trimethylation of lysine 9 on histone H3, emerged as the second most predictive regional feature. For the EM based model, the frequency of the TGTC end motif was identified as the most influential sequence feature, indicating that end‐motif composition contributes to morphological discrimination (Figure 4C). The FLEN model ranked the frequency of 138‐bp fragments as the most informative length‐derived feature among the evaluated bins (Figure 4D).

To further explore the biological relevance of these informative features, beside adjusted methylation level of sample, the TE H3K9me3 peaks donated the second prediction power to TE model which predicted embryo quality, and ∼18% genes on those peak region can be annotated on promoter in Figure 4E. Having obtained annotations to nearest genes, functional enrichment analysis are performed to identify predominant biological themes among these genes, Figure 4F shows those genes correlated with promoters were mainly enriched on cell adhesion and glucuronidation. During embryonic development, cell adhesion activities are the key mechanisms driving cell compaction and the formation of tight junctions, and these processes jointly affect the establishment and stability of blastocysts [32, 33]. The glucuronic acid pathway is an important branch of glucose catabolism, and its core functions include the synthesis of hyaluronic acid (HA), chondroitin sulfate and other mucopolysaccharides [34]. A study showed that HA improves the bovine embryo quality by regulating the DNA methylation and expression patterns of the focal adhesion pathway [35].

Overall, these results demonstrate that embryo‐derived cfDNA harbors biologically meaningful signals reflective of developmental competence, which can be effectively captured through comprehensive feature extraction and machine learning approaches. The identification of critical genomic regions, epigenetic markers, and sequence motifs highlights the molecular foundations underlying embryo morphological quality.

### 2.5 Association Between Key Feature Scores and Embryo Clinical Outcomes

**FIGURE 5:**
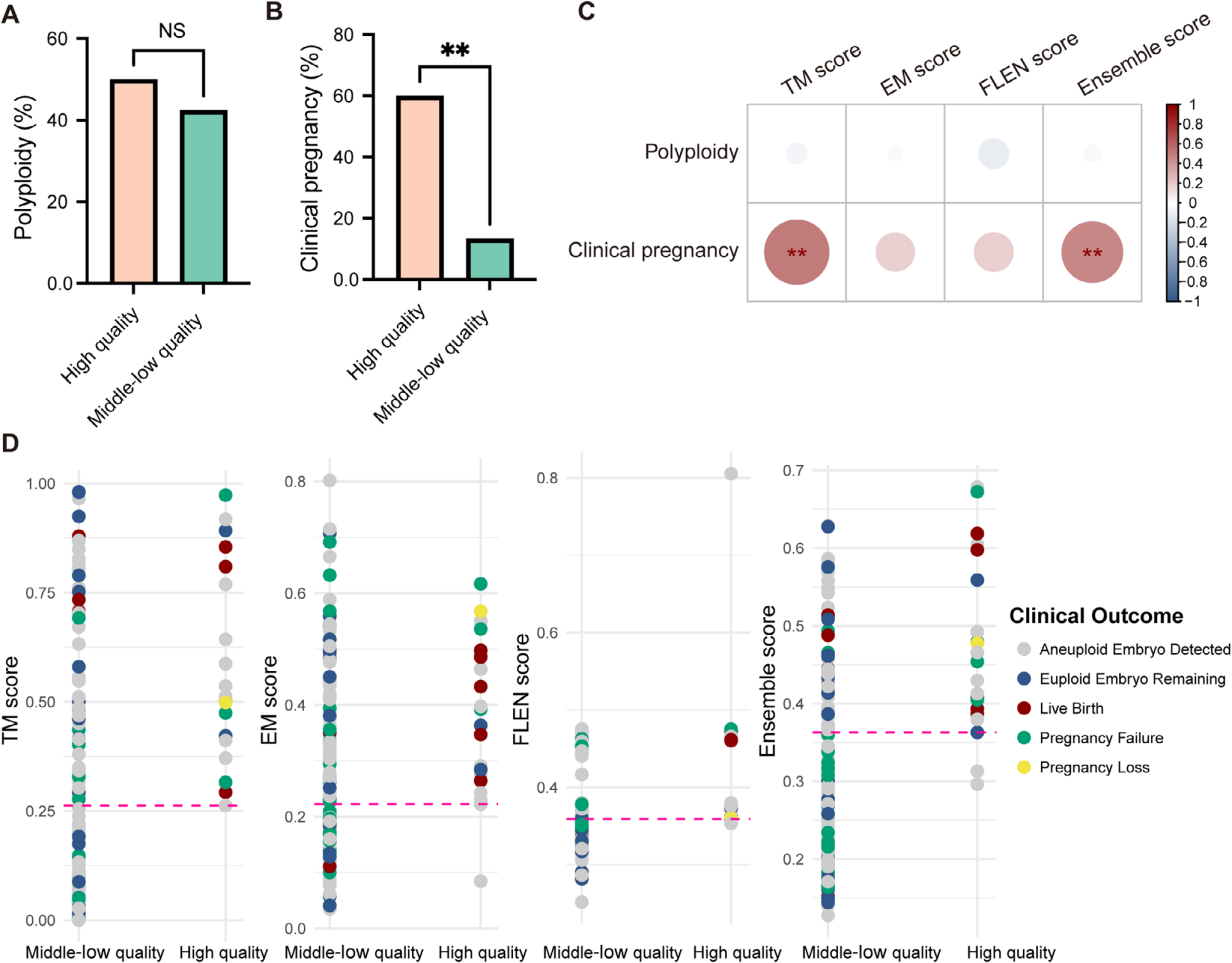
Embryo quality assessment and feature scores. (A) Barplot showing euploid rate of high quality group and middle‐low quality group. (B) Barplot showing clinical pregnancy rate of high quality group and middle‐low quality group. (C) The correlation between clinical indicators and four machine learning derived scores, where the size of the dots represents the size of Spearman's correlation coefficient. (D) Overview of enrolled participants and the clinical outcomes of their embryos. The *Y*‐axis represented the score values derived from machine learning of the embryo. Dashed line represents the cutoff value of the corresponding feature's ROC curve. ^*^ *p* < 0.05; ^**^ *p* < 0.01; ^***^ *p* < 0.001.

Our study included 154 embryos. A total of 39 euploid embryos and 1 aneuploid embryo underwent selective single embryo transfer. Clinical data showed 30 cases of pregnancy failure, 1 cases of miscarriage, and 9 live births (Table S3). The clinical outcomes were consistent with the predictions of morphological assessment, showing that the clinical pregnancy rate in the high quality group was significantly higher than that in the middle‐low quality group. However, there was no significant difference in the overall euploid rate between the two groups (Figure 5A, B). The trend shown in Figure 5A is consistent with the established morphology and euploidy, but the limited data volume precludes the determination of statistical differences.

To evaluate whether the feature scores contain biological signals that are meaningful for embryo quality assessment, we reviewed the clinical outcomes of embryos at different score levels. We analyzed the correlations between four key feature scores and euploid rate and clinical pregnancy rate. Notably, all key feature scores were positively correlated with the clinical pregnancy rate, especially the TM score and Ensemble score (Figure 5C). It indicated that when the feature score is higher, embryos are more likely to achieve better clinical outcomes, that is, higher embryo quality.

We further plotted the distribution relationship between four key feature scores and all embryo outcomes. It can be seen that the four scores showed a similar trend, that is, the higher the score, the more likely to achieve clinical pregnancy and live birth (Figure 5D). Under the same morphological assessment results, we can select embryos with higher scores for transplantation.

Overall, the above results further confirm the robustness of the NICE framework for embryo quality assessment in real‐world clinical applications. These feature scores can provide more information for clinical decisions in embryo selection.

## 3 Discussion

In this study, we developed NICE, a two‐step framework for non‐invasive embryo assessment based on cfDNA extracted from SECM. By first enriching embryo‐derived cfDNA fragments to minimize maternal contamination and subsequently applying a machine learning based strategy to classify embryo morphological quality, NICE presents a promising, non‐invasive, and damage‐free approach for embryo selection. Through extensive feature extraction and model interpretability analyses, we identified critical genomic regions, methylation signatures, and end motifs associated with embryo quality, highlighting the biological relevance of cfDNA‐derived signals. Morphological assessment fails to provide biological information regarding their genetic status, while also being subject to inter‐operator variability. We hope to develop a more standardized and automated system for evaluating embryo quality, as well as a system that can provide more information about genetic biological data. Our research results show that there is a significant positive correlation between the feature scores obtained based on machine learning algorithms and the clinical pregnancy rate. This means that embryos with higher scores tend to lead to more ideal clinical outcomes. These findings suggest that NICE may serve as a quantitative cfDNA‐based indicator by integrating genetic and epigenetic information captured from SECM‐derived cfDNA. By converting multiple cfDNA‐derived signals into a standardized score, NICE provides an additional molecular reference for identifying embryos with greater viability. If further validated in larger, independent, and prospectively designed cohorts, this quantitative assessment strategy may help refine embryo prioritization in ART and support efforts to improve pregnancy outcomes.

Previous studies have explored the use of niPGT by analyzing cfDNA released into the culture medium, primarily focusing on detecting embryo ploidy status. However, challenges such as maternal DNA contamination and limited cfDNA quantity have hindered its widespread clinical application. Approaches including genetic amplification, parental genotyping, and bioinformatic deconvolution have been proposed to improve accuracy [20, 36]. Meanwhile, fragmental features such as cfDNA fragment size, end motifs have shown great potential in tissue‐of‐origin deconvolution and cancer detection [37, 38]. Yet, their applicability in niPGT remains unclear. Building on this, our work shifts the focus from purely chromosomal analysis to a comprehensive, multi‐feature evaluation. By integrating methylation profiles, fragment distribution, end motif patterns, and regional copy number variations, NICE offers a scalable machine‐learning‐based framework for predicting embryo viability. The reduced performance observed when models were trained on unfiltered cfDNA further supports the importance of embryo‐derived signal enrichment before downstream feature extraction and quality assessment.

Despite the promising results, several limitations should be acknowledged. First, the sample size remains relatively modest, which may limit the generalizability of the trained models to broader clinical populations. The limited number of high‐quality embryos also constrains the stability of model evaluation, particularly in estimating performance under imbalanced class distributions. Additionally, while we demonstrated robust performance using cross‐validation, external validation on independent datasets from different laboratories or clinical centers is necessary to confirm the robustness and reproducibility of the NICE framework. Second, although the unfiltered‐read sensitivity analysis supports embryo‐derived cfDNA enrichment as an important preprocessing step, the current read‐enrichment threshold should be regarded as a predefined analytical choice. Its optimal setting, robustness across datasets, and potential association with embryo‐related covariates such as developmental stage, morphology, or fragmentation will require validation in larger datasets and systematic multi‐threshold analyses.

As our study did not mandate the donation of spent embryo culture medium and relied on voluntary participation, the enrolled sample size was limited, precluding comprehensive clinical outcome tracking. A sufficient number of participants is essential to validate that embryos classified as high‐quality by our model indeed correlate with better clinical outcomes, such as higher implantation and live birth rates. Therefore, future studies will aim to expand the clinical dataset by incorporating samples from multiple centers, covering a broader range of embryo qualities, developmental stages, and patient backgrounds. In parallel, the current feature extraction approach still relies heavily on prior biological knowledge, which may introduce certain biases. With the advancement of large foundation models pre‐trained on extensive human methylome datasets [39], it is becoming increasingly feasible to automatically extract and integrate sequence‐derived information into embryo‐level representations. Leveraging such models could enable a more comprehensive and unbiased characterization of cfDNA signals, potentially leading to improved prediction of embryo viability and live birth outcomes.

## 4 Methods

### 4.1 Data Preparation

This study utilized three primary data sources: (i) Single‐cell bisulfite sequencing (scBS‐seq) data for training the DECENT‐plus model were obtained from a previously published study [20]. We collected single‐cell methylomes corresponding to two cell types: TE and ICM. On average, each cell contributed approximately 8.4 GB of sequencing data, covering about 10.8 million CpG sites. Detailed information regarding the training data is provided in Table S1. (ii) Methylation profiles of PBs were derived from a published study by Yuan et al. [40]. (iii) SECM data and cumulus cells were sourced from a previously published study by Chen et al. [15]. This dataset consisted of 194 PGT‐A blastocysts along with their corresponding culture media samples and 12 cumulus samples. The cell‐type methylomes used for DECENT‐plus training and the SECM samples used for downstream embryo‐quality classification were derived from distinct sample sets, with no overlap at the embryo, patient/couple, or biological‐sample level. We graded embryos according to the Gardner morphological blastocyst grading system, where AA blastocysts were considered high quality, B^*^ blastocysts (AB, BA, and BB) were classified as middle quality, and the remaining C^*^ blastocysts (BC and CB) were classified as low quality. We tracked the outcomes of all of these embryos up to January, 2026. Clinical outcomes of the transplant cycle were defined as follows: clinical pregnancy: visualization of at least one gestational sac by ultrasound 30 days after embryo transfer; live birth: the birth of one or more live infants; and pregnancy loss: ectopic pregnancy or abortion before 28 weeks of gestation.

Preprocessing steps included adapter trimming, removal of amplification primers, and filtering of low‐quality bases. Subsequently, R2 reads containing more than three unmethylated CH sites, along with their corresponding R1 reads, were discarded. Clean reads were aligned to the human reference genome (hg19) using BS‐Seeker2 v2.1.1 (https://github.com/BSSeeker/BSseeker2) [41]. Unaligned reads were re‐mapped using the local alignment mode, and alignments within microhomologous regions with low confidence were removed. PCR duplicates were eliminated using Picard Tools v1.119 (https://broadinstitute.github.io/picard/) [42].

During DECENT‐plus model training, increasing the amount of training data substantially prolonged the training time. To balance resource constraints and model performance, we expanded the training dataset compared to DECENT (which utilized 15 million reads from two cell types). In DECENT‐plus, we randomly sampled 7.5 million reads from each of the four cell types (cumulus cells, PBs, TE, and ICM), resulting in a total of 30 million reads for training. Reads derived from cumulus cells and PBs were labeled as maternal‐origin contamination, while reads from TE and ICM cells were labeled as embryo‐origin cfDNA. DECENT‐plus was trained to predict the origin of each individual read.

### 4.2 Training and Evaluation of DECENT‐Plus

Compared with the original DECENT method, DECENT‐plus retains the same core read‐level contamination‐scoring framework but introduces three main updates. First, the input length per read was extended from 66 to 132 bp, allowing the model to use the full read sequence and methylation context rather than truncated inputs. Second, the maternal‐contamination training class was expanded from cumulus cell derived reads only to a combined cumulus cell and polar body read set, better reflecting the maternal DNA sources present in SECM. Third, the training set was increased from 15 to 30 million reads to improve model stability. Although this increase required more computational resources, it allowed the model to capture longer sequence contexts and incorporate more methylation information, ultimately improving classification accuracy and overall model performance.

Each read was encoded by simultaneously integrating both sequence and methylation features. Cytosines identified as methylated were labeled as mC. The sequence was then one‐hot encoded across five possible states: A, T, C, G, and mC. Each base was represented as a 5D binary vector (e.g., A as [1, 0, 0, 0, 0], T as [0, 1, 0, 0, 0]). As a result, the input data for DECENT‐plus had a shape of N × 132 × 5, where N denotes the number of reads.

For model training, we randomly sampled 30 million reads and split them into a training set and a test set at an 8:2 ratio. Reads derived from TE/ICM cells were labeled as 0 (embryo‐derived), whereas reads from cumulus cells and PBs were labeled as 1 (maternal contamination). The encoded reads were passed sequentially through a 1D convolutional layer, a multi‐head attention layer, a bidirectional LSTM (Bi‐LSTM) layer, another 1D convolutional layer, and a MLP layer. Finally, a sigmoid activation function was applied at the output layer to produce a probability score between 0 and 1, representing the likelihood that a read originated from maternal contamination.

The model was trained using the binary cross‐entropy loss function and optimized with the Adam optimizer, with an initial learning rate of 0.01 and a batch size of 128. Training was performed for 30 epochs. After training, model performance was evaluated on the test set using accuracy and AUROC metrics to assess the classification capability of DECENT‐plus.

### 4.3 Reads Score Thresholding for Ambiguous Read Exclusion in DECENT‐Plus Evaluation

To evaluate the robustness of DECENT‐plus under varying levels of classification certainty, a score thresholding strategy was implemented to exclude ambiguous reads. For a given threshold t∈{0.2,0.3,0.4,0.5} reads with DECENT‐plus scores falling within the symmetrical interval (*t*, 1 − *t*) were defined as ambiguous and excluded from the evaluation. This filtering approach partitioned the data into high‐confidence subsets: reads with scores in [0,  *t*] were classified as embryo‐derived, while those in [1 − *t*,  1] were classified as maternal‐derived. The AUC was subsequently calculated for each threshold level using only the remaining high‐confidence reads. This stepwise increase in *t* allowed for a systematic assessment of how narrowing the exclusion interval, and thus including more lower‐confidence reads, impacted model performance. When *t*  =  0.5 the exclusion interval collapsed to a single point, equivalent to evaluating the entire, unfiltered test set. This sensitivity analysis was designed to identify the optimal confidence range for reliable downstream identification of embryo‐derived reads.

### 4.4 Estimation of Maternal Contamination Levels

Using the trained DECENT‐plus model, we assigned to each cfDNA read in the SECM samples a probability score (s) representing its likelihood of originating from maternal contamination. The overall maternal contamination level (r) for each SECM sample was then estimated by aggregating the predicted scores (s) across all reads. The maternal contamination rate is computed using the following formula:

$$r=argmax_{r}\;\prod_{i=1}^{N}\left(r\times s_{i}+\left(1-r\right)\times \left(1-s_{i}\right)\right)$$

where *N* is the total number of reads in the SECM sample and *s_i_* is theDECENT‐plus‐predicted probability of the *i^th^* read being of maternal origin.

### 4.5 Feature Extraction

All features were derived from bisulfite sequencing reads after maternal‐origin filtering. For each SECM sample, DECENT‐plus generated a read‐level maternal‐contamination probability score, which was used to enrich high‐confidence embryo‐derived cfDNA reads. Reads with scores below 0.2 were retained and used to calculate the TM, GWM, EM, TRN, FLEN, and CNV feature sets for downstream embryo‐quality assessment.

#### 4.5.1 TM

DNA methylation patterns in specific genomic regions are known to play critical roles in regulating embryonic development. We selected 24 embryo development–related regions and 22 genome‐related functional regions from previous studies (Table S2). For each region i, the average methylation level was calculated as

$$Methylation\;level=\frac{Number\;of\;methylated\;Cs}{Total\;number\;of\;Cs}$$

In addition to these regional features, a sample‐level methylation rate was calculated as the genome wide ratio of methylated cytosines to all observed cytosines. To correct for maternal contamination, the contamination proportion (*r_contam_*) for each sample was estimated using DECENT‐plus, and a linear regression model was fit to relate the sample‐level methylation rate to the contamination proportion: *M*_sample_ =  α + β*r*_contam_ +  ε. The adjusted sample‐level methylation was then defined as *M*_adjusted_ = *M*_sample_  − β*r*_contam_, representing the expected methylation under zero contamination; values were constrained to the [0, 1] interval. The adjusted sample‐level methylation was included as an additional component of the TM feature set.

#### 4.5.2 GWM

While region‐specific methylation captures local signals, genome wide patterns may reflect broader epigenetic states associated with embryo viability. To this end, we divided the genome into 661 non‐overlapping 5 Mb bins and computed the average methylation level for each bin, serving as the GWM features.

#### 4.5.3 EM

End motifs of cfDNA fragments are associated with nucleosome protection and nuclease cutting preferences. To capture these sequence‐level features, we extracted the first 4 nucleotides at the 5′ end of each read. The frequency of each of the 256 possible 4‐mer combinations was calculated, resulting in a 256‐dimensional EM feature vector per sample.

#### 4.5.4 TRN

The genomic distribution of cfDNA fragments can reflect chromatin accessibility and degradation patterns. We counted the number of reads aligned to each of the 661 non‐overlapping 5 Mb bins. To account for differences in sequencing depth across samples, the read counts were normalized using *z*‐score transformation (mean‐centered and scaled by standard deviation) within each sample. These normalized values were used as the TRN features.

#### 4.5.5 FLEN

Fragment length reflects nucleosome structure and fragmentation bias in cfDNA. For each sample, the relative frequency of each read length was computed, yielding a compositional feature vector whose components are non negative and sum to one.

#### 4.5.6 CNV

CNV features were derived using the Ginkgo platform (version 3.0.0) [28]. Briefly, the genome was partitioned into 5541 bins, and the number of reads mapped to each bin was counted. Ginkgo then applied GC bias correction and segmentation (e.g., using Circular Binary Segmentation) to estimate the relative copy number in each bin. The normalized copy number values across all bins were used as CNV features for downstream classification.

### 4.6 Machine Learning for Embryo Quality Classification

We applied trained DECENT‐plus to filter out maternal reads in each sample. Specifically, we retained only reads with a DECENT‐plus score below 0.2, corresponding to high‐confidence embryonic reads. This enrichment procedure was used to reduce ambiguous maternal contributions and retain embryo‐derived cfDNA signals for downstream feature extraction and embryo‐quality assessment. After filtering, we assessed the number of remaining embryonic reads in each sample. Samples with insufficient read coverage for reliable feature extraction were excluded from further analysis. Ultimately, 154 samples with adequate embryonic read content were retained for downstream feature extraction and subsequent embryo morphological quality classification using machine learning models.

To classify sample quality, we first constructed single‐feature models based on the aforementioned individual features. The resulting sample‐level feature matrices were paired with morphology‐derived embryo‐quality labels and used as inputs for supervised machine‐learning classification. Samples with the morphological label “AA” were labeled as 1 (high quality), while samples with other morphological labels were labeled as 0, forming a binary classification task. Given the significant class imbalance (high quality samples constituted only 14% of the dataset), we employed the Synthetic Minority Over‐sampling Technique (SMOTE) from the Python package imblearn (version 0.13) to address this issue. SMOTE generates synthetic samples for the minority class by interpolating between each minority sample and its k‐nearest neighbors (default k = 5), effectively balancing the class distribution and mitigating the performance degradation caused by class imbalance.

For each feature type, features containing missing values were excluded, and the remaining features were standardized to zero mean and unit variance using StandardScaler (scikit‑learn). For compositional features (FLEN and EM), a centered log‑ratio (CLR) transformation was applied prior to modeling to mitigate closure‑induced spurious correlations, to embed the data in Euclidean space compatible with standard learning algorithms, and to improve comparability across samples; zero entries were handled by adding a small pseudocount before transformation. Subsequently, high‑variance features were selected using univariate ANOVA (f_classif); the number of selected features k was determined in a dataset‑specific manner based on performance within the training data. PCA was then applied for dimensionality reduction and linear feature combination, with the number of components chosen to retain 95% of the cumulative explained variance.

We employed five machine learning algorithms, MLP, SVC, XGB, RF, and LR, to build the models. During training, we utilized 5‐fold cross‐validation and optimized hyperparameters using Optuna (version 4.0) with n_trials = 30. The hyperparameter search space for each model was defined as follows: MLP, number of neurons in each hidden layer, selected from [(50,), (100,), (50, 50)]. L2 regularization term, sampled logarithmically between 0.0001 and 0.1; SVC, kernel type, selected from [“linear”, “sigmoid”, “poly”, “rbf”], regularization parameter C (0.1–10); XGB, number of estimators (50–200), max depth (2–10), learning rate (0.0001–0.1), subsample ratio of the training instances, sampled uniformly between 0.1 and 1.0, minimum sum of instance weights required in a child, sampled as an integer between 1 and 10; RF, number of estimators (30–100), max depth (2–20); LR, regularization parameter C (0.1–10).

The best‐performing model for each feature type, as determined by the ROC, top 3 of them single models were selected and ensembled by AUC‐weighted averaging of their prediction scores. Performance was evaluated by ROC within each fold of five‐fold cross‐validation was selected for stacking with weighted ensembl.

### 4.7 Interpretable Analysis of Feature Importance

We analyzed feature importance for each model constructed from individual feature types to gain insights into the model's decision‐making process. For tree‐based models like Random Forest, we directly extracted feature importance (feature_importances_) from each cross‐validation fold and computed the mean importance across folds. For Logistic Regression, we used the absolute values of the feature weight parameters as a measure of feature importance. For models such as MLP, SVC, and XGB, we employed the KernelExplainer from the Python package SHAP (version 0.47.0) to calculate Shapley values [43], which quantify the marginal contribution of each feature to the model's predictions. Shapley values, rooted in cooperative game theory, assign responsibility for the model's predictions to each feature by evaluating its contribution across all possible feature combinations, providing a robust framework for interpretability at both the global and individual sample levels. To ensure consistency, we averaged Shapley values across all samples and folds of the cross‐validation.

### 4.8 Genomic Region Annotation and Methylation Level Visualization

To explore the biological relevance of the top‐ranked features, we performed GO enrichment analysis using the clusterProfiler package (version 2.1.4) in R. The enrichment analysis was conducted with a significance threshold of *p* < 0.05, and *p*‐values were adjusted for multiple testing using the Benjamini‐Hochberg procedure. For the visualization of methylation levels in genomic regions, we utilized the ChIPseeker package (version 1.38) in R.

### 4.9 Statistical Analysis

Statistical analysis was performed using Python 3.11 and R software (version 4.4.2, R core team). Wilcox test was used to compare the continuous data, while chi‒square tests or Fisher's exact tests were used to compare categorical data. The Kolmogorov‐Smirnov test was implemented using the ks_2samp (version 1.15.2) in Python. The Spearman correlation coefficient was calculated using the R package psych (version 2.4.3). All *p* values were two‐sided, with *p* < 0.05 considered to indicate statistical significance.

## Author Contributions

Y.C., P.Z. and J.Q. conceived and supervised the project. X.Z. and P.Z. designed the algorithm; X.Z. implemented the algorithm and performed the computations. S.D. performed the clinical data analysis. All authors analyzed the data and interpreted the results. X.Z. and S.D. drafted the initial manuscript, which was revised and approved by all authors.

## Conflicts of Interest

The authors declare no competing interests.

## Code Availability

Our source code and models are publicly available at https://github.com/XueyaZhou‐124/NICE

## Supporting information




**Supporting File 1**: advs77327‐sup‐0001‐SuppMat.xlsx.

**Supporting File 2**: advs77327‐sup‐0002‐SuppMat.docx.

## Data Availability

The datasets supporting this study were derived from publicly available resources and are summarized in Supplementary Table S1. For Stage 1, training data include single‐cell methylation profiles of polar bodies (PB) from Yuan et al. [40], cumulus cells from Chen et al. [15], andinner cell mass (ICM) along with trophectoderm (TE) cells from (GEO: GSE81233). For Stage 2, the SECM datasets were obtained from Chen. et al. [15]. All data origins and accession details are fully documented in the cited literature and supplementary materials.
