# Supplementary material for: NICE: A Two‐Step Non‐Invasive Framework for Embryo cfDNA Read Enrichment and Quality Assessment
Source: Adv Sci (Weinh). 2026 Aug 24;13(63):e77327. doi: 10.1002/advs.77327 (PMC13502268; doi:10.1002/advs.77327)
Supplement: Supplementary file 1 [file ADVS-13-e77327-s001.docx]

# **Supplementary Information**

# Supplementary Notes

# Supplementary Note 1. Training data of the Stage 1 DECENT-plus model in the NICE pipeline.

# Supplementary Note 2. TM panel design for embryo classification.

# Supplementary Note 3. Validation of contamination-corrected methylation dispersion patterns.

# Supplementary Note 4. **Classification performance without stage1 DECENT-plus enrichment.**

**Supplementary Figures**

**Supplementary Figure 1. Training and evaluation of DECENT-plus.**

**Supplementary Figure 2. Comparison of methylation dispersion across embryo quality groups.**

Supplementary Figure 3. Exploratory analysis of TRN and CNV features.

Supplementary Figure 4. Classification performance of different feature sets without **DECENT**-plus enrichment.

Supplementary Figure 5. ROC curves from the without-SMOTE sensitivity analysis.

**Supplementary Tables**

**Supplementary Table 1. Training data for DECENT-plus.**

**Supplementary Table 2. Predefined genomic target region.**

**Supplementary Table 3. Clinical outcome for samples in this study.**

Supplementary Note 1. Training data of the Stage 1 DECENT-plus model in the NICE pipeline

To train DECENT-plus to discriminate embryo-derived cfDNA from maternal cfDNA, we used single-cell scBS-seq data from four representative cell types: trophectoderm (TE), inner cell mass (ICM), cumulus cells, and polar bodies (PBs). Although generated for methylation profiling, scBS-seq reads preserve native sequence-level characteristics and fragmentation patterns, enabling their use for read-level origin classification. The sample list and basic sequencing statistics are provided in Supplementary Table 1.

For each cell type, preprocessed and mapped scBS-seq reads (after the same quality control and alignment procedures as described in the main Methods) were pooled across all available single-cell samples. From each pooled cell-type–specific dataset, 7.5 million reads were randomly sampled at the read level to construct the training dataset. TE and ICM reads were used to represent embryo-derived cfDNA, whereas cumulus cells and PBs reads were used as maternal cfDNA surrogates.

The resulting training dataset thus consisted of 15 million embryo-derived reads and 15 million maternal-derived reads, which were used to train DECENT-plus in Stage 1 of the NICE pipeline. All data source information is summarized in Supplementary Table 1.

**Supplementary Note 2. TM panel design for embryo classification.**

We provide the full list of 46 target-region methylation (TM) features in Supplementary Table 2, together with their genomic annotations and corresponding statistics, including effect size, p-value, and adjusted p-value. Each TM feature corresponds to a predefined genomic region represented as a BED interval, for which the average DNA methylation level was computed per sample as described in the Methods.

Among the 46 TM features, five exhibited statistically significant differences between high- and middle-low quality embryos after multiple testing correction and are presented in Figure 3C in the main text. The remaining features did not reach statistical significance in this cohort but are reported here for completeness and to facilitate future validation in independent datasets.

# **Supplementary Note 3.** Validation of contamination-corrected methylation dispersion patterns

To evaluate whether the contamination-corrected cfDNA methylation levels in SECM reflect biologically meaningful patterns, we compared our results with biopsy-based measurements reported by Guo et al.^1^. In Guo et al., embryos were stratified into AA (high quality), B* (middle quality), and C* (low quality) groups according to morphological assessment, and global methylation levels were derived directly from embryo biopsy samples. Their data showed that high quality embryos exhibited more concentrated methylation values, whereas middle and low quality embryos displayed increased dispersion.

We applied the same quality grouping to our cohort and examined the distribution of contamination-corrected sample-level methylation values derived from SECM cfDNA. After linear regression-based adjustment for maternal contamination (Methods; Figure 3A), the dispersion pattern across quality groups closely recapitulated that reported by Guo et al.. Corrected methylation levels in high quality embryos were tightly clustered, while middle and low quality embryos showed progressively greater variability (Supplementary Figure 1).

To formally test differences in dispersion, we used Bartlett’s test for homogeneity of variances across the three quality groups, both for the biopsy data^1^ and for our contamination-corrected SECM cfDNA methylation. The significant heterogeneity of variances in both datasets supports that the corrected cfDNA-based methylation feature captures variability patterns consistent with direct embryo biopsy measurements.

# **Supplementary Note 4. **Classification performance without Decent-plus enrichment****

To evaluate how much the Stage 1 enrichment (DECENT-plus) contributes to the overall classification performance, we trained the same Stage 2 models directly on cfDNA from low-contamination SECM samples, without applying read-level enrichment. Specifically, we restricted this analysis to samples with an estimated net ratio ≤ 30%, where the net ratio is a previously proposed non-deep learning-based estimate of maternal contamination level (as described in the original SECM study and in Methods). We then recomputed all feature categories (TM, GWM, FLEN, TRN and EM) from the unfiltered cfDNA. The same model types, feature definitions, and cross-validation scheme as in the main analysis were used.

At the overall model level, the two-stage NICE pipeline provided a clear performance gain: the best-performing ensemble model improved from an AUC of 0.708 in the raw low-contamination setting to 0.812 with Stage 1 enrichment (Figure 3G). This supports the use of read-level embryo enrichment as the primary strategy.

However, the impact of Stage 1 enrichment was feature-dependent (Supplementary Figure 2). For TM, the AUC increased from 0.449 without enrichment to 0.806 with enrichment, indicating that removal of maternal reads markedly enhances the signal-to-noise ratio for TM. In contrast, a few feature categories showed comparable or slightly better performance without enrichment. For example, TRN feature achieved an AUC of 0.63 in the raw low-contamination setting versus 0.551 with enrichment. These features may rely on more global cfDNA properties and thus remain informative even when maternal cfDNA is retained, or they may partly benefit from the additional complexity present in mixed cfDNA.

Overall, these analyses indicate that while Stage 1 enrichment consistently improves the best achievable performance and is particularly beneficial for embryo-specific methylation features, certain non-embryo-specific or more global features are less dependent on enrichment and can perform similarly, or occasionally slightly better, when computed directly from low-contamination cfDNA. This feature-dependent behavior supports our two-stage design as the main analysis, while also demonstrating that the Stage 2 framework is reasonably robust in the absence of explicit read-level enrichment.

**Supplementary Figure 1. Training and evaluation of DECENT-plus.** (**A)** Training and validation accuracy curves over 30 epochs, showing model convergence. **(B)** Confusion matrix of the test set results. The model achieved a global accuracy of 67.54% in distinguishing between maternal (Cumulus cells/PBs) and embryonic (TE/ICM) reads.

**Supplementary Figure 2. Comparison of methylation dispersion across embryo quality groups.** (**A)** Contamination corrected cfDNA methylation levels in SECM from this study, grouped using the same embryo quality categories. **(B)** Global methylation levels of high quality (AA), middle quality (B*), and low quality (C*) embryos based on biopsy data reported by Guo et al. Differences in variance among the three groups were assessed using Bartlett’s test for homogeneity of variances.

**Supplementary Figure 3. Exploratory analysis of TRN and CNV features between embryo quality groups. (A)** Genome-wide distribution of total read number (TRN) across 5 Mb genomic bins. The x-axis indicates genomic bin positions ordered by chromosome, and the y-axis represents z-score–normalized TRN values within each sample. Each point corresponds to the mean TRN of a genomic bin across samples, colored by embryo morphological quality group. **(B)** Principal component analysis of CNV profiles derived from embryo-origin cfDNA in SECM samples. Each point represents one sample, colored by embryo quality group.

**Supplementary Figure 4. Classification performance of different feature sets without DECENT-plus enrichment. (A)** Area under the ROC curve (AUC) for embryo quality classification using individual feature categories (TM, GWM, FLEN, TRN and EM) when Stage 2 models are trained directly on low-contamination SECM cfDNA without DECENT-plus–based read enrichment (“raw low-contamination” setting). Each bar represents the mean AUC across cross-validation folds. **(B)** AUCs of ensemble models combining the three best-performing feature categories from panel A using weighted voting.

**Supplementary Figure 5. ROC curves from the without-SMOTE sensitivity analysis.**

ROC curves for all Stage-2 feature sets evaluated under the same cross-validation framework without SMOTE oversampling. The Stage-2 models were trained and evaluated without synthetic minority-sample generation while keeping the feature sets and evaluation procedure otherwise unchanged. The target methylation feature set (TM) retained the highest discrimination performance in the without-SMOTE setting (AUROC = 0.816), while the other feature sets showed lower AUROC values (GWM = 0.590, EM = 0.633, FLEN = 0.611, TRN = 0.533, CNV = 0.535, Concat = 0.629). These results indicate that the primary methylation-based signal was not driven solely by SMOTE-based class rebalancing.

# Reference

1.	Li, G. *et al.* Genome wide abnormal DNA methylome of human blastocyst in assisted reproductive technology. *J. Genet. Genomics* **44**, 475–481 (2017).
